# Leveraging a people-centred approach to combat antimicrobial resistance in Africa

**DOI:** 10.1186/s41182-025-00863-w

**Published:** 2025-11-28

**Authors:** Yusuff Adebayo Adebisi, Wuraola Akande-Sholabi, Nafisat Dasola Jimoh, Hajar Lali, Kehinde Asake Adebowale, Amal Ouachhou, Kenneth Chukwuebuka Egwu, Loureen Valyne Nachibwede, Deborah Oluwaseun Shomuyiwa, Ahishakiye Gilbert, David Olpengs, Noah Sesay, Iyiola Olatunji Oladunjoye

**Affiliations:** 1 Global Health Focus, Abuja, Nigeria; 2 Department of Clinical Pharmacy and Pharmacy Administration, Faculty of Pharmacy, University of Ibadan, Ibadan, Nigeria; 3 Global Health Focus, Kigali, Rwanda; 4 Faculty of Medicine and Pharmacy, Hassan II University, Casablanca, Morocco; 5 Department for Global Public Health, Karolinska Institutet, Stockholm, Sweden; 6 Faculty of Medicine and Pharmacy, Mohammed V University, Rabat, Morocco; 7 Department of Pharmacy, University of Nigeria Teaching Hospital, Ituku-Ozalla, Enugu, Nigeria; 8 School of Public Health, Jomo Kenyatta University of Agriculture and Technology-JKUAT, Juja, Kenya; 9 College of Public Health, University of Georgia, Athens, GA USA; 10 College of Medicine and Health Sciences, University of Rwanda, Kigali, Rwanda; 11 Amref Health Africa, Nairobi, Kenya; 12 Ministry of Health of Sierra Leone, Ola During Children’s Hospital, Freetown, Sierra Leone; 13 Department of Microbiology, Faculty of Life Sciences, University of Ilorin, Ilorin, Nigeria

**Keywords:** Antimicrobials, Antimicrobial resistance, People-centred approach, Public health, Africa

## Abstract

Antimicrobial resistance (AMR) poses a severe and growing threat to public health in Africa, disproportionately affecting marginalised and vulnerable populations across communities and health systems. Current responses often prioritise technical measures, such as stewardship programmes and surveillance systems, with insufficient attention to the socioeconomic and cultural realities that drive resistance. AMR cannot be addressed in isolation, as its emergence and spread are closely linked to poverty, inadequate education, gender inequality, poor governance, limited access to healthcare, clean water, sanitation, and diagnostics, as well as weak supply chains for essential medicines. This commentary advocates for a people-centred approach to AMR that addresses the social determinants of health and fosters inclusive, community-driven solutions. Strengthening primary healthcare systems and improving access to affordable, quality-assured antimicrobials and diagnostics must be prioritised to empower both healthcare providers and patients. Local stakeholders are essential for raising awareness, promoting behaviour change, and ensuring cultural relevance through meaningful community engagement. Marginalised populations, including those in underserved regions or disproportionately exposed to infection due to displacement, disability, or comorbidities, should be central to the co-creation of AMR strategies. Embedding AMR initiatives within universal health coverage reforms, expanding preventive measures, such as vaccination, and tackling systemic challenges are also crucial for reducing antibiotic dependence and building equitable health systems. A coordinated, multisectoral response that connects human, animal, and environmental health, grounded in equity, community ownership, and interdisciplinary collaboration, is essential for sustainable AMR control efforts that leave no one behind.

## Introduction

Antimicrobial resistance (AMR) is a global public health challenge, with Africa bearing a disproportionate share of the burden. A recent study estimated that, in 2019 alone, the WHO African Region experienced approximately 1.05 million deaths associated with bacterial AMR, with 250,000 deaths directly attributable to resistant infections [1]. The highest fatal burden was linked to lower respiratory infections (48%), bloodstream infections (22%), and intra-abdominal infections (10%) [1]. The leading pathogens responsible for these deaths included *Streptococcus pneumoniae*, *Klebsiella pneumoniae*, *Escherichia coli*, and *Staphylococcus aureus*, with third-generation cephalosporin-resistant *Klebsiella pneumoniae* and meticillin-resistant *Staphylococcus aureus* being prevalent in large parts of the region [1].

AMR interventions in Africa have primarily relied on technical measures, such as antimicrobial stewardship programmes, surveillance systems, and regulatory frameworks [2–4]. Although stewardship initiatives have achieved some success in tertiary hospitals, their impact is constrained by limited diagnostics, resource shortages, and inadequate workforce training [3, 5]. Likewise, implementation of the Global Antimicrobial Resistance and Use Surveillance System (GLASS) remains uneven across countries, with persistent challenges in data collection, funding, and laboratory capacity [5]. Regulatory frameworks also struggle to address informal antibiotic markets, leaving vulnerable populations dependent on unregulated sources [6]. As a result, AMR efforts remain largely facility-based, with minimal engagement of community settings where much antibiotic use occurs. This gap, combined with the inconsistent integration of equity considerations in national AMR action plans [2], highlights the need for more people-centred strategies.

While technical solutions are essential to combating AMR, they often overlook the lived realities of the people most affected. In rural and underserved areas where healthcare access is limited, overcrowding is common, sanitation is poor, and health literacy is low, communities are disproportionately impacted by resistant infections [7]. Many people in these settings depend on informal or unregulated providers, which contributes to the misuse and overuse of antibiotics [7]. These social, economic, and infrastructural challenges are closely interlinked, and addressing them in isolation is unlikely to succeed. Awareness campaigns are also unlikely to succeed when affordable and quality-assured healthcare services remain out of reach. This persistent gap between technical responses and everyday realities perpetuates inequities and undermines the sustainability of AMR control efforts. A truly people-centred approach that prioritises marginalised populations and confronts the systemic barriers they face is, therefore, essential [8–10]. Without such comprehensive reform, AMR strategies risk failing those most in need.

**Fig. 1:**
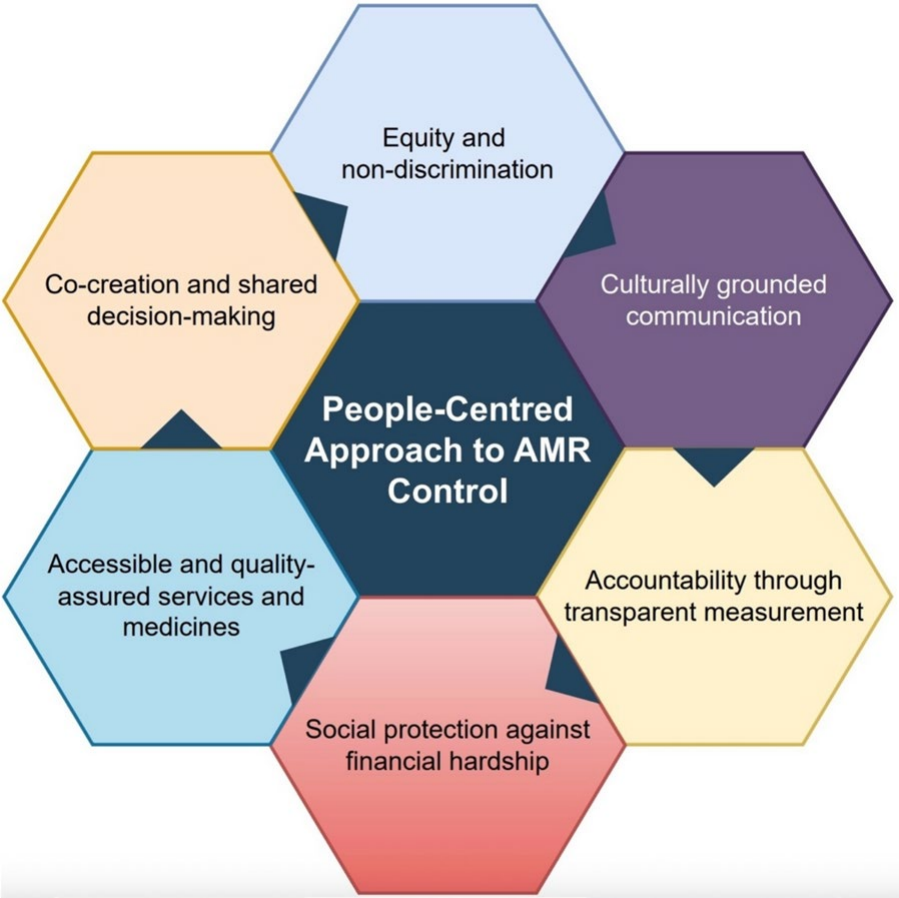
Conceptual synthesis of a people-centred approach to antimicrobial resistance (AMR) control

These realities underline the need for a paradigm shift in AMR control, moving from institution-focused interventions to approaches that place people at the centre of prevention and response. Building on global guidance but advancing it further, this paper proposes a conceptual synthesis of a people-centred approach to AMR that positions individuals, families, and communities as active partners across prevention, access, diagnosis, and treatment. The proposed framework identifies six core elements: equity and non-discrimination; co-creation and shared decision-making; accessible and quality-assured services and medicines; culturally grounded communication; social protection against financial hardship; and accountability through transparent measurement (Fig. 1). While these principles resonate with WHO’s emphasis on people-centred stewardship and national action plan implementation [11], they are here articulated as an integrated framework tailored to the realities of AMR control in Africa. This synthesis underpins the analysis that follows and provides the foundation for developing AMR responses that genuinely reflect people’s needs and lived experiences.

## Systemic and people-centred factors in addressing AMR

**Fig. 2:**
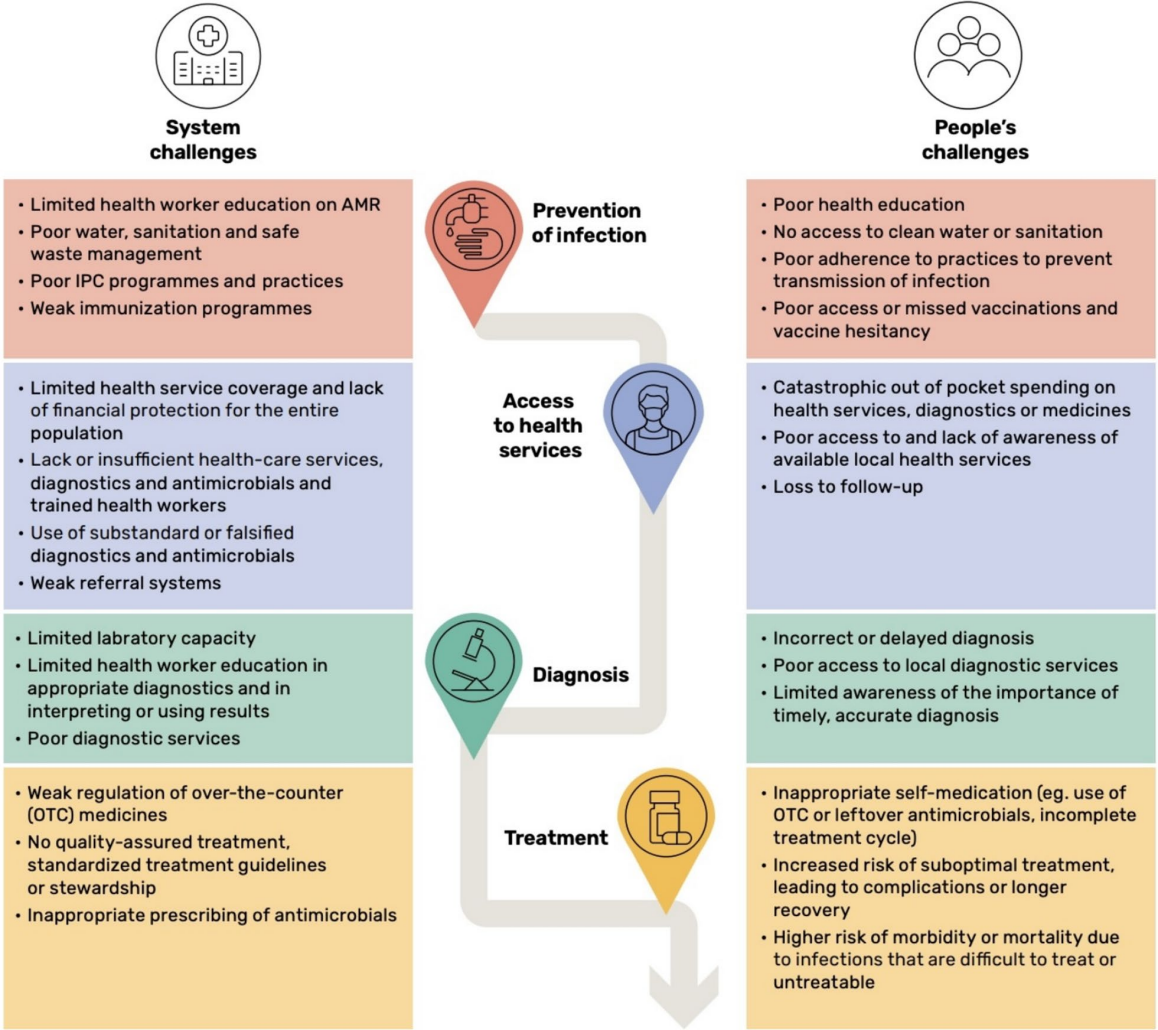
Interconnected systemic and people-centred barriers across the AMR response pathway. Source: WHO [11]

AMR cannot be adequately curbed without considering the broader social determinants of health that influence how individuals and communities interact with healthcare systems. Poverty remains one of the most significant drivers of AMR in Africa [2, 12]. In many rural and underserved areas, communities often rely on unregulated drug vendors or purchase antibiotics over-the-counter without prescriptions due to the lack of formal medical services [13, 14]. This widespread availability of antimicrobials outside regulated systems leads to misuse, overuse, and the eventual emergence of resistant pathogens. Moreover, financial constraints often push individuals to seek cheaper alternatives or partial doses, further compounding the problem [14]. A case example from Nigeria illustrates how practices such as medicine rationing, incomplete doses based on patient affordability, and mixing antimicrobials from different or same drug classes are common among Patent and Proprietary Medicine Vendors (PPMVs), further driving inappropriate antimicrobial use and resistance [14]. Figure 2 (reproduced from WHO) for a visual summary of the systemic and people-centred challenges in addressing AMR [11].

Poor health literacy also contributes to antimicrobial misuse [15]. However, it must be understood alongside the structural and economic barriers that shape people’s choices. Many individuals know they should not share antibiotics or stop treatment early, yet financial hardship, weak health systems, and the high cost of medicines often make completing a full course impossible. In such settings, so-called “inappropriate use” frequently reflects constrained access rather than ignorance or negligence [16, 17]. A systematic review of 24 cross-sectional studies from countries including Nigeria, Kenya, Ethiopia, South Africa, Ghana, Tanzania, Uganda, and Zambia identified low educational levels, poor knowledge of AMR, and attitudes toward antibiotic use as major contributors to misuse [18]. In addition, a review of AMR education and awareness initiatives in the WHO African Region found that 92 studies from 19 Member States documented widespread suboptimal knowledge, poor attitudes and practices, and extensive self-medication [15]. These findings underline the need for context-specific interventions that combine health education with broader systemic reforms ensuring that affordable, quality-assured medicines and reliable healthcare services are available to support appropriate antibiotic use. In a people-centred AMR strategy, health literacy initiatives should empower communities without shifting the burden of response onto them. Programmes delivered in local languages and grounded in cultural contexts can promote informed decision-making, while public health campaigns should emphasise preventive measures such as vaccination to reduce infection risk and reliance on antibiotics.

Access to clean water and sanitation is another essential determinant of health that intersects with AMR. Poor sanitation and inadequate water supply are linked to the spread of infectious diseases, leading to increased antibiotic use [19]. In sub-Saharan Africa, nearly 400 million people still lack access to safe drinking water, and many suffer from waterborne diseases [20], which often prompt inappropriate antibiotic treatments. A recent ecological study evaluating the relationship between community water and sanitation access and the global burden of antibiotic resistance found that increased access to improved drinking water and sanitation was associated with lower abundance of antibiotic resistance genes (ARGs) in human fecal metagenomes [21]. This association was more pronounced in urban areas compared to rural areas, highlighting the critical role of infrastructure in mitigating AMR in low- and middle-income countries. The study, which analysed 1589 metagenomes from 26 countries, revealed that the mean abundance of ARGs was highest in Africa compared to Europe, North America, and the Western Pacific [21]. A people-centred approach to AMR involves addressing basic infrastructure deficits by ensuring communities have access to clean water, effective waste management, and promoting awareness and implementation of infection prevention and control practices at the household level to reduce infection transmission and the reliance on antimicrobials.

In addition, the lack of robust healthcare infrastructure in many African countries limits access to accurate diagnostics, resulting in empirical or inappropriate antibiotic prescriptions [22]. A study conducted in the Kumbo East and Kumbo West Health Districts in Cameroon found that antibiotics were often prescribed for conditions such as malaria or when diagnoses were uncertain, reflecting a significant reliance on broad-spectrum antibiotics in the absence of adequate diagnostic tools [23]. The study reviewed over 30,000 prescriptions and reported an antibiotic prescription rate of 36.7%, with amoxicillin being the most commonly prescribed antibiotic (29.9%) [23]. Notably, all prescribed antibiotics were broad-spectrum, and empirical prescribing was influenced by factors, such as limited use of laboratory results and patient turnover rates [23]. Rural areas, in particular, suffer from a shortage of diagnostic tools and trained healthcare workers, leading to a reliance on broad-spectrum antibiotics [22]. This misuse fails to address the actual cause of infection and also accelerates the development of resistant pathogens. Strengthening healthcare infrastructure, especially in marginalized regions, is key in implementing effective AMR strategies.

Although AMR manifests as a biological process, its drivers and impacts are fundamentally social, shaped by inequities in living conditions, access to care, and the structural determinants of health (Table 1). Recognizing AMR as a social problem requires addressing the interconnected social determinants of health that exacerbate its spread [24]. Poverty, lack of education, inadequate healthcare infrastructure, and poor access to clean water and sanitation all directly or indirectly contribute to the misuse of antibiotics and the rise of resistance, particularly in low- and middle-income countries [7, 12, 25, 26, 28]. To effectively combat AMR, it is essential to adopt a comprehensive approach that considers these underlying factors. Efforts must include improving healthcare access, enhancing public health education, especially in marginalized communities, and ensuring infrastructure development for clean water and sanitation.

**Table 1:** Social and structural factors contributing to antimicrobial resistance

Factor	Impact on AMR	Illustrative evidence
Poverty	• Limits access to healthcare, driving self-medication and purchase of non-regulated antimicrobials • Incomplete antibiotic courses due to cost promote resistance • Low-income settings depend heavily on informal drug sellers	The HATUA Consortium study in Kenya, Tanzania, and Uganda found that financial barriers, inconvenient healthcare systems, and unregulated antibiotic access fuelled non-adherence and self-medication [25]
Limited education	• Reduces awareness of appropriate antibiotic use (e.g., using antibiotics for viral infections, stopping early) • Weak understanding of infection prevention increases antibiotic reliance	A meta-analysis of 39 African studies (*n* = 18,769) reported pooled knowledge, attitude, and practice scores of 55.3%, 46.9%, and 51.0%, respectively, highlighting major educational gaps [26]
Globalisation and mobility	• International travel, migration, and trade accelerate cross-border spread of resistant bacteria	A review of 238 studies found increasing travel-related spread of quinolone-resistant and multidrug-resistant *Salmonella* spp. from Asia and Africa [27]
Behavioural norms and practices	• Sharing or rationing antibiotics and mixing with traditional remedies reflect limited access and distrust of formal care • Social norms framing antibiotics as “cure-alls” perpetuate misuse	In Nigeria, Patent and Proprietary Medicine Vendors dispense antibiotics in partial doses (“cut medicine”) and mix classes without prescriptions, practices rooted in affordability constraints and weak regulation [14]
Household location	• Rural and underserved communities face poor access to regulated antimicrobials and diagnostics • Weak sanitation and hygiene systems heighten infection spread and antibiotic demand	A study in rural Uganda showed reliance on informal drug shops, poor health literacy, and limited diagnostics driving inappropriate antibiotic use; the authors called for integrated literacy, access, and stewardship reforms [28]
Weak governance and regulation	• Poor enforcement of drug policies allows proliferation of counterfeit and substandard antibiotics • Weak supply chain systems contribute to inappropriate distribution and use	A systematic review and meta-analysis across 23 studies from Sub-Saharan Africa found that 69% of antibiotic requests at community drug retail outlets were dispensed without a prescription, reflecting widespread regulatory gaps and poor enforcement of antibiotic control laws [6]

## Role of community engagement in AMR containment

Community engagement is a cornerstone of the people-centred approach, as it translates high-level strategies into actionable, localised solutions while ensuring cultural relevance and sustainability [11]. It can be understood along a continuum, from basic information sharing and consultation to deeper forms of collaboration and co-production. At its simplest, engagement involves providing communities with information and opportunities to express their views. At higher levels, co-production or co-creation goes further by positioning communities as equal partners who jointly design, implement, and evaluate interventions. Clarifying this distinction is critical for AMR policy: while engagement increases awareness and dialogue, co-production redistributes power and ensures that interventions genuinely reflect community priorities.

One of the critical gaps in the current approach to AMR is the lack of genuine community involvement in shaping policies and interventions [8, 29]. Communities in Africa have a wealth of knowledge about local health practices, which, if harnessed effectively, can be a powerful tool in combating AMR. Local health practices, including the use of traditional medicine, are deeply rooted in many African cultures and have been used for generations. Integrating traditional medicine practitioners into the fight against AMR could expand the reach of AMR awareness campaigns and also make these interventions more acceptable and trusted within communities [30]. For example, traditional healers often serve as the first point of contact for many people in some rural areas, especially where formal healthcare systems are weak or inaccessible [30].

Evidence from the Community-led Solutions to Antimicrobial Resistance (COSTAR) project and a community dialogue intervention in Mozambique further demonstrate the importance of engaging communities in health initiatives [31, 32]. In Mozambique, trained local facilitators led structured dialogues in villages about schistosomiasis prevention. Community members jointly identified risk behaviours, discussed feasible protective measures, and agreed on local action plans. This participatory process, rather than a top-down campaign, led to significant improvements in knowledge and preventive practices [32]. Such approaches illustrate how culturally tailored dialogue can build collective responsibility and trust, improving adherence to AMR-related initiatives. Furthermore, religious leaders and teachers should be included as key stakeholders in AMR initiatives. Religious leaders hold significant influence in many African communities and have historically played critical roles in health campaigns, such as the fight against HIV/AIDS [33, 34]. Their ability to mobilize large groups and deliver trusted messages makes them invaluable allies in AMR education and advocacy.

Similarly, schoolteachers can instill knowledge about AMR in younger generations, fostering a culture of responsible antibiotic use from an early age. The e-Bug programme demonstrated that training educators through interactive workshops significantly improved their confidence and ability to teach AMR and infection prevention [35]. With 95% of participants rating the training positively, this approach highlights the potential of empowering educators to embed AMR awareness into curricula, shaping responsible health behaviours in future generations [35]. By involving these respected community figures, AMR campaigns can achieve greater reach, cultural resonance, and long-term sustainability.

A people-centred approach emphasises co-creation, where communities are not passive recipients of health interventions but active participants in the design and implementation of AMR strategies. Engaging community leaders, community health workers, and other local stakeholders in dialogue about AMR can help tailor interventions to the specific needs and challenges of different regions. A recent survey of 35 civil society organisations (CSOs) working in 37 African countries highlights their critical but underutilized role in AMR prevention and control [36]. The survey revealed that while CSOs are engaged in advocacy, accountability, and service delivery aligned with Africa CDC’s AMR Framework, many lack structured strategies and robust monitoring mechanisms. Only 20% reported using metrics to monitor their activities, and 80% had no AMR-specific strategies, underlining the need for capacity building and training. Strengthening CSOs and community organisations to co-create AMR interventions with communities can improve alignment with national action plans and regional strategies, thereby enhancing sustainability and impact [36].

Practical examples of co-creation include involving communities in message development, testing communication materials for clarity and cultural relevance, and deciding on preferred media formats, whether storytelling, radio, theatre, or digital platforms. This differs from conventional awareness campaigns, because the choice of content and delivery stems from the community itself. Evidence from participatory health communication research shows that co-created messages are more likely to be trusted and acted upon [31]. Furthermore, involving communities in the monitoring and evaluation of AMR interventions fosters a sense of ownership and accountability, which is critical for the sustainability of these programmes. When communities see themselves as partners in the fight against AMR, rather than mere beneficiaries, they are more likely to take responsibility for ensuring that practices such as antimicrobial stewardship are followed [11]. This bottom-up approach also allows for the incorporation of indigenous knowledge, which can provide valuable insights into alternative treatments and preventive measures [37], potentially reducing the reliance on antibiotics.

In addition, community engagement is essential in addressing the social stigma often associated with illness and antibiotic use. In some African societies, individuals may hide infections or refuse treatment out of fear of being ostracized, which can lead to the spread of resistant pathogens [38]. Similarly, vaccine hesitancy must be tackled through culturally sensitive education and awareness programs, as improving vaccination rates can significantly reduce the burden of infections and the subsequent reliance on antibiotics [11]. By engaging communities in open discussions about AMR and destigmatizing treatment, local health systems can create environments where people feel comfortable seeking medical help when needed [39]. This, in turn, reduces the misuse of antibiotics and encourages timely and appropriate treatment. Ultimately, fostering long-term commitment to fighting AMR at the grassroots level requires policies that are not only top-down but also bottom-up, with input from all stakeholders. This inclusive approach ensures that AMR interventions are culturally relevant, socially acceptable, and more likely to succeed in the diverse contexts across Africa.

## Empowering healthcare workers

Healthcare workers are at the forefront of the battle against AMR, yet many in Africa face significant challenges due to a lack of resources, training, and infrastructure. In many rural and underserved areas, healthcare providers often lack the diagnostic tools and lab reagents needed to accurately identify infections, leading to the over-prescription of broad-spectrum antibiotics [40]. This contributes to the development of AMR, which can also undermine the trust between patients and healthcare systems. Therefore, a people-centred approach must prioritise investing in the capacity building of healthcare professionals, ensuring they have the tools, knowledge, and resources necessary to manage antibiotic use and resistance effectively.

Training programmes focused on AMR stewardship are essential, particularly for healthcare workers in primary care settings [41]. These programmes should also incorporate community-oriented training that equips healthcare workers to address cultural and socioeconomic factors that influence antibiotic misuse. Engaging healthcare workers in understanding local beliefs and practices related to antibiotics can improve their ability to communicate effectively with patients. These programmes should also include modules on appropriate antimicrobial prescribing practices, diagnostic tools to distinguish between bacterial, viral, parasitic, and fungal infections, and alternative treatment options for common infections. For instance, rapid diagnostic tests can assist healthcare workers in identifying the specific cause of an infection, such as differentiating between bacterial and viral origins or detecting parasitic infections like malaria. Similarly, enhanced diagnostic capabilities for fungal infections, such as point-of-care testing for candidiasis or cryptococcosis, can minimise unnecessary antimicrobial use and improve treatment outcomes. However, such diagnostic tools are often unavailable or underused in low-resource settings.

While investing in infrastructure that supports accurate diagnosis is key to reducing AMR in these regions [42], the financial and political realities cannot be ignored. Many African health systems operate under chronic underfunding, with competing priorities such as maternal health, infectious disease control, and non-communicable diseases absorbing limited budgets. As a result, diagnostic expansion is often viewed as aspirational rather than immediately achievable. Addressing this requires phased, cost-effective strategies, such as integrating AMR diagnostics into existing laboratory networks, leveraging public–private partnerships, and advocating for domestic financing mechanisms and donor alignment. Strengthening political commitment through evidence of cost-savings from early diagnosis and rational prescribing can also help secure sustained investment.

Moreover, frontline healthcare workers must be equipped with the skills to engage patients in meaningful discussions about the risks of antibiotic misuse. Patient education initiatives have demonstrated their effectiveness in increasing awareness of appropriate antibiotic use and reducing unnecessary prescription rates. For example, a systematic review of 18 high-quality studies assessed the impact of various forms of patient education on antibiotic use and expectations [43]. The studies included interventions, such as videos, public health campaigns, leaflets, posters, mixed approaches, and presentations. Active learning methods such as interactive videos and community-based public health campaigns showed significant improvements in patient knowledge and reduced expectations for antibiotics, whereas passive approaches, such as posters and standalone leaflets, had minimal or no effect. These findings emphasise the importance of integrating interactive and engaging educational tools into healthcare settings to maximize their impact [43]. This underlines the need to equip healthcare providers with communication tools and strategies to effectively educate patients about rational antibiotic use. However, these interventions must also be accompanied by supportive systems [44], such as affordable access to essential medicines, functioning referral mechanisms, and supervision, so that healthcare workers are not placed in the impossible position of advising rational use where the system itself cannot deliver appropriate alternatives. Healthcare workers should also be included in feedback mechanisms to share real-world challenges and successes, enabling continuous improvement of stewardship programmes. Incorporating their insights ensures that interventions remain adaptive and aligned with community needs.

Healthcare workers addressing AMR are under immense psychological pressure, often grappling with feelings of helplessness, burnout, and stress as they face an invisible enemy armed with diminishing therapeutic options [45]. This mental health toll is exacerbated in resource-limited settings, where inadequate staffing, lack of diagnostic tools, and high patient loads are prevalent. Integrating mental health support into stewardship programmes, such as resilience training, peer support networks, and access to counselling services, is critical [45]. These initiatives will safeguard the well-being of healthcare providers and also enhance their capacity to deliver effective, patient-centred care, thereby strengthening the overall response to AMR [46, 47].

## Policy and regulatory reforms for people-centred and equitable AMR control

Policy and regulatory frameworks are vital in the fight against antimicrobial resistance, but for them to be effective, they must be designed with the realities of people’s lives at their core. In many African countries, weak enforcement of regulations governing the sale and distribution of antibiotics allows the proliferation of counterfeit, substandard, and improperly prescribed medicines [48], exacerbating the AMR crisis. A regulatory environment that fails to monitor the pharmaceutical supply chain and hold violators accountable creates a market where dangerous and ineffective drugs can easily reach consumers, increasing the risk of drug resistance. However, these weaknesses often reflect deeper systemic and economic realities. Regulatory agencies in many low- and middle-income countries operate with limited funding, fragmented mandates, and dependence on donor-driven projects rather than stable domestic investment. In such settings, enforcement capacity is constrained not by lack of intent but by lack of fiscal space, skilled personnel, and political prioritisation of regulatory reform.

Access to antibiotics is important, as is their regulation. A people-centred approach to AMR requires that policy reforms go beyond drafting regulations on paper and instead focus on building robust systems to enforce them effectively. For instance, governments must strengthen drug regulatory agencies, ensuring they have the capacity to monitor the quality and distribution of medicines. This includes increasing the transparency of drug supply chains, from manufacturers to pharmacies, to prevent counterfeit and substandard antibiotics from entering the market [48]. Strengthening the capabilities of regulatory bodies will require investing in technology and human resources, particularly in areas, such as border control, laboratory testing of medicines, and pharmacy inspections. However, expecting resource-poor governments to suddenly build comprehensive regulatory systems is unrealistic. A pragmatic approach involves risk-based regulation, focusing scarce resources on high-risk products and regions, and regional cooperation through pooled procurement, shared testing facilities, and harmonised standards, as seen in initiatives by the African Medicines Agency (AMA). These incremental steps can improve regulatory reach without imposing unaffordable costs.

Moreover, many African countries face significant challenges with unregulated informal markets where antibiotics are sold without prescriptions [6]. In these markets, antibiotics are often dispensed without appropriate medical guidance, contributing to the misuse of these drugs. Yet informal vendors persist not merely, because regulation is weak, but because they fulfil real social and economic functions. In communities where healthcare is costly or distant, local drug sellers offer accessibility, credit, and trust. Similarly, vendors may sell counterfeit or broken-up antibiotic packs, because demand exists from consumers who cannot afford a full course. Any reform that ignores these incentives risks driving the trade further underground. Reforming these informal sectors is a complex but necessary task. Policymakers must develop strategies that encourage the formalization of these markets, creating incentives for vendors to register with regulatory authorities. This could include micro-licensing schemes, training on safe dispensing, and linking vendors to formal supply chains, so that they can sell quality-assured medicines at controlled prices. For example, a micro-licensing model tied to accredited training and quality-assured supply can shift informal sellers toward safer dispensing while maintaining local access, and risk-based inspection can prioritise hotspots with high rates of inappropriate antibiotic sales. Community outreach programmes that educate both vendors and consumers about the risks of selling and using antibiotics without prescriptions are essential to this effort.

At the same time, regulatory frameworks must be flexible enough to ensure that access to essential medicines is not hindered for those who genuinely need them [13]. Many marginalized populations, especially those in rural and underserved areas, face barriers to accessing healthcare, including geographical distance, cost, and lack of healthcare facilities. When healthcare is inaccessible, individuals often turn to informal markets or self-medication as a more affordable and convenient alternative [49]. A people-centred policy reform would address these barriers by improving access to healthcare services and making quality antibiotics affordable and available through legitimate channels. Affordability remains the central tension in AMR regulation: patients may fully understand the need to complete a prescribed course but lack the means to buy it. Tackling this requires policies that reduce out-of-pocket spending, such as including antibiotics on essential medicine reimbursement lists, subsidising key drugs, or enabling bulk procurement to lower retail prices. Partnerships with generic manufacturers and pooled regional purchasing mechanisms can also reduce costs. Without addressing affordability, enforcement efforts alone will penalise the poor rather than protect them.

National policies should define safe self-care pathways for common, self-limiting infections by (i) issuing pharmacy-led triage and “watch-and-wait” protocols, (ii) limiting over-the-counter availability to non-antibiotic symptomatic treatments while prohibiting non-prescription systemic antibiotics, (iii) mandating standard pack sizes and clear front-of-pack labelling on treatment duration, and (iv) enabling pharmacist follow-up or delayed prescriptions where appropriate. Coupled with affordability measures, these steps preserve access while curbing inappropriate antibiotic use.

The socioeconomic factors driving AMR in humans are complex, but a focused understanding of these complexities can guide the development of more effective interventions. The emergence and proliferation of AMR are influenced by an interplay of factors, such as gender, living conditions, education, healthcare accessibility, ineffective governance, human migration, conflict, climate change, agricultural practices, and environmental pollution [50]. Policies that acknowledge these interconnections and address their combined impact are more likely to succeed in achieving their objectives [11]. Recognising this complexity also requires that policymakers balance deterrence with empathy, understanding why individuals or vendors act as they do, rather than assuming non-compliance stems from ignorance. Economic insecurity, weak health insurance coverage, and limited provider availability all shape behaviour within pharmaceutical markets. Effective AMR regulation must, therefore, be accompanied by broader social protection and universal health coverage efforts that make formal care a viable option for all.

Furthermore, policymakers need to take into account the socioeconomic realities that influence healthcare-seeking behaviours. In regions where poverty is widespread, the costs associated with travel to healthcare facilities, consultation fees, and diagnostics can be prohibitive. A policy framework that promotes access to primary healthcare services closer to where people live, such as through mobile clinics, telemedicine, or community health worker programmes, can reduce the reliance on self-medication and the purchase of antibiotics from informal sources [51].

Finally, regulatory frameworks must also prioritise the development of antimicrobial stewardship programmes that align with people’s everyday experiences. Stewardship programmes should be designed with input from healthcare workers, patients, and community leaders, ensuring that they are practical and culturally sensitive. These programmes must include guidelines for healthcare providers on appropriate antibiotic prescribing practices, as well as monitoring and evaluation systems to track the effectiveness of AMR interventions. Engaging communities in the creation of these guidelines is key for fostering public trust and ensuring that stewardship programmes are well-received and adhered to at the grassroots level. Ultimately, people-centred AMR policy and regulatory reform must strike a balance between tightening controls on antibiotic use and ensuring equitable access to quality medicines. Financing these reforms will require blended approaches that combine domestic health insurance coverage, catalytic donor support, and partnerships with generic manufacturers to keep essential antibiotics within reach for all income groups.

## Integrating AMR efforts into universal health coverage

The pursuit of universal health coverage (UHC) in Africa provides an opportunity to embed antimicrobial resistance control within wider health system reforms. UHC aims to ensure that everyone can access quality health services without financial hardship [51, 52]. Integrating AMR strategies into this agenda helps ensure that essential medicines, diagnostics, and infection prevention services are available to all, particularly those most at risk of exclusion. It also aligns with the One Health framework, which recognises that effective AMR control depends on addressing human, animal, and environmental health together [53]. However, in many low- and middle-income countries, antimicrobial stewardship and AMR surveillance remain only partially reflected in national UHC strategies, often implemented as vertical programmes rather than as core components of health-system strengthening [54, 55]. This fragmentation limits policy coherence and constrains progress toward equitable antibiotic access and sustainable resistance containment.

Evidence from 103 countries shows that limited UHC coverage is strongly associated with inadequate AMR capacities [54]. Expanding national insurance schemes to include essential antibiotics and diagnostics would reduce out-of-pocket spending and encourage full treatment adherence [55, 56]. Within UHC reforms, governments can strengthen pharmaceutical supply chains, regulate antibiotic pricing, and integrate AMR surveillance indicators into routine health information systems [57]. Linking these indicators to UHC reporting would promote policy coherence across ministries and improve accountability.

Integrating AMR into UHC also reinforces the central role of primary healthcare. Well-equipped local facilities can provide early diagnosis, appropriate prescribing, and infection prevention, reducing reliance on informal drug markets [58]. UHC financing mechanisms, such as pooled procurement, community health worker programmes, and telemedicine, offer cost-effective routes to expand access without major new infrastructure [55, 57]. Development partners can complement these efforts through catalytic investments in laboratory capacity and digital surveillance.

Finally, embedding AMR control in UHC strengthens prevention. UHC reforms that prioritise vaccination, sanitation, and hygiene directly reduce infection rates and, by extension, antibiotic demand [58, 59]. Aligning AMR and UHC goals, therefore, creates a foundation for equitable, affordable, and sustainable health systems capable of containing resistance while ensuring that no one is left behind.

## The role of research and innovation

Research and innovation are central to a sustainable response to AMR in Africa. While global studies have expanded understanding of AMR dynamics, local evidence remains limited. A people-centred research agenda should prioritise the social, economic, and behavioural drivers of resistance across African contexts. Understanding how factors such as informal healthcare provision, antibiotic affordability, and local treatment practices shape antimicrobial use can inform policies that are both effective and equitable [14]. For example, studies are needed on how community drug sellers and traditional healers, who often serve as first points of care, can be meaningfully integrated into formal health systems through training, regulation, and collaboration.

Robust surveillance and data infrastructure are also crucial. A recent review found that AMR data are unavailable for 42.6% of African countries, while resistance to commonly used antibiotics such as amoxicillin and trimethoprim exceeds 80% in some settings [60]. This highlights the need for continent-wide investment in laboratory capacity, diagnostic standardisation, and interoperable data systems that link human, animal, and environmental health sectors. Strengthening these systems will enable early detection of resistance patterns and guide rational antimicrobial use.

Innovation in diagnostics and therapeutics must also reflect Africa’s resource realities. Affordable rapid diagnostic tests that distinguish between bacterial and viral infections can reduce empirical antibiotic prescribing. Parallel research into novel treatments, such as bacteriophage therapy, vaccines, and plant-based antimicrobials, could lessen reliance on conventional antibiotics if these innovations are made locally accessible [61]. Governments and regional bodies can accelerate their uptake by streamlining regulatory approval processes and creating incentives for local production of quality-assured antimicrobials and diagnostics [48].

Building a research ecosystem that supports such innovation requires sustained investment in human capital, cross-disciplinary collaboration, and equitable partnerships. Funding mechanisms should prioritise African-led studies that address context-specific challenges and translate findings into policy and practice. A coordinated regional research strategy, aligned with Africa CDC’s AMR framework, would help avoid duplication, foster capacity building, and ensure that innovations ultimately serve communities most affected by resistance. Table 2 summarises key areas of research that can inform people-centred AMR interventions in Africa, including diagnostics, behavioural research, pharmaceutical regulation, and innovation financing.

**Table 2:** Key research priorities for a people-centred AMR response in Africa

Research area	Guiding questions
Healthcare access and equity	How can health systems reduce barriers to diagnosis and treatment, particularly for rural and low-income populations?
Informal and community health systems	What strategies can integrate informal providers and traditional healers into safe antibiotic stewardship?
Diagnostics and surveillance	What low-cost diagnostic tools and surveillance models are most feasible in resource-limited settings?
Behavioural and cultural factors	How do local beliefs, norms, and information channels influence antibiotic use and resistance?
Pharmaceutical regulation and supply chains	How can national and regional systems prevent substandard, falsified, or unprescribed antibiotic sales?
Innovation and technology	How can digital tools and artificial intelligence improve AMR monitoring and access to quality medicines?
Vulnerable populations	How can AMR policies better protect marginalised groups affected by poverty, displacement, or disability?
Community ownership and engagement	What participatory approaches ensure that AMR interventions are accepted, monitored, and sustained locally?

## Marginalized and vulnerable populations in a people-centred AMR approach

A people-centred approach to AMR in Africa acknowledges that marginalized and vulnerable populations face distinct challenges that are compounded by systemic inequalities. Poor access to healthcare, clean water, and proper sanitation disproportionately affects these groups, exposing them to a higher risk of infectious diseases and frequent antibiotic use [11]. Yet, a recent equity-focused analysis of AMR National Action Plans (NAPs) across 14 West African countries revealed that, while all countries adopt the One Health framework, few explicitly recognise or address the needs of vulnerable populations, such as refugees, people with disabilities, incarcerated persons, or those living in extreme poverty [2]. Most NAPs only mention healthcare workers and rural populations, with little evidence of measurable equity indicators or strategies to ensure inclusion of disadvantaged groups. This gap highlights the urgent need to embed equity considerations within AMR policies and ensure that interventions reach those at highest risk.

For instance, neonates, children, immunocompromised individuals, and people with comorbidities are particularly vulnerable to infections due to their weakened immune systems [11]. Refugees, many of whom are displaced by conflict and live in camps across sub-Saharan Africa, also face these risks. The lack of adequate healthcare infrastructure, clean water, and proper sanitation in camps like those in Sudan, South Sudan, and the Democratic Republic of the Congo significantly heightens their risk of infectious diseases [62]. These conditions result in the overuse of antibiotics, often without proper medical guidance [62]. Refugees and forcibly displaced persons may resort to informal healthcare providers, where antibiotic misuse is rampant [63–65]. By actively involving these communities in co-designing AMR interventions, relevant stakeholders can develop culturally relevant programmes that empower refugees with the knowledge needed to manage antibiotics appropriately.

For people living with disabilities (PLWD) in Africa, accessibility to healthcare is often restricted by inadequate infrastructure, such as poorly equipped medical facilities or limited transportation options, especially in rural areas [66, 67]. Many PLWD in African countries rely on caregivers or informal healthcare providers, which increases their vulnerability to antibiotic misuse, especially in regions where there is limited access to diagnostic tools or trained healthcare personnel. In countries, such as Nigeria and Kenya, disability-inclusive healthcare is still in its infancy, often leaving PLWD underserved [68]. A people-centred approach focuses on involving PLWD and their caregivers in the creation of AMR strategies that address these unique challenges. For example, improving access to healthcare services by incorporating mobile clinics, telemedicine, or community outreach programmes can bridge the gap between PLWD and formal healthcare systems. In addition, education on antibiotic use that is delivered through accessible formats, such as local language materials or visual aids, ensures that PLWD are informed and can make decisions that prevent the misuse of antibiotics [10].

Incarcerated people across Africa, particularly in countries, such as South Africa, Zambia, and Ethiopia, often live in overcrowded, unsanitary conditions where infectious diseases are common, and healthcare services are insufficient [69]. The over-prescription of antibiotics, often used as a blanket approach to control infections in prisons where diagnostic facilities are limited, exacerbates the AMR crisis [70–72]. A people-centred AMR response in prisons requires systemic reform, improving healthcare infrastructure and sanitation while engaging incarcerated people and prison healthcare workers in the development of antibiotic stewardship programmes. By ensuring dialogue and including incarcerated people in the design and implementation of these interventions, healthcare providers can ensure that the programmes reflect the realities of prison life, leading to more effective and sustainable solutions. This approach shifts the narrative from top-down enforcement to one that includes incarcerated people in the effort to address AMR, promoting both their health and dignity.

The intersection of AMR and mental health is another critical, yet often overlooked, aspect of the crisis [45]. Fear-based messaging around AMR can inadvertently fuel anxiety and stress among the general population, particularly in communities already grappling with healthcare insecurity. Healthcare workers, particularly in resource-constrained settings, also face significant mental health burdens due to the increasing prevalence of AMR. The emotional toll of managing resistant infections with limited resources, coupled with the fear of potential treatment failures, often leads to burnout, anxiety, and depression. This psychological strain affects their capacity to provide optimal care, creating a vicious cycle that undermines efforts to combat AMR. Moreover, people living with mental health disorders are uniquely vulnerable to the consequences of AMR. Limited access to mental health services, coupled with societal stigma, may leave these individuals more reliant on informal healthcare providers. This further elevates their risk of encountering resistant infections. A people-centred approach to AMR must include mental health support, not only for healthcare workers but also for communities, ensuring that those living with mental health conditions are not left behind in efforts to tackle this crisis.

## Recommendations

To operationalise a people-centred approach to AMR containment in Africa, the following actions are proposed:

1. Co-create community-led and equity-oriented AMR interventions with civil society organisations, faith-based groups, schools, grassroots leaders, and traditional healers to improve awareness, ownership, and behavioural change at the local level.
2. Adopt micro-licensing, training, and formal supply linkages for informal antibiotic vendors, combined with risk-based regulatory inspections to reduce unsafe dispensing practices.
3. Include essential antibiotics and basic diagnostics in national health insurance benefit packages and regulate retail prices to improve affordability and adherence.
4. Integrate AMR surveillance indicators into UHC and health information systems, and publish annual AMR performance scorecards to strengthen accountability across ministries.
5. Scale up community health worker and telemedicine platforms to expand early diagnosis, appropriate prescribing, and infection prevention in rural and underserved communities.
6. Finance national and regional laboratory networks through pooled procurement mechanisms, shared testing capacity, and catalytic donor support to enhance diagnostic access.
7. Target WASH and vaccination investments toward high-burden districts as part of joint AMR–UHC implementation plans.
8. Strengthen the mental health and well-being of frontline healthcare workers within antimicrobial stewardship programmes through psychosocial support and workload management measures.
9. Embed inclusion and equity in AMR education and public health interventions to ensure that marginalised and vulnerable groups are meaningfully reached.
10. Establish sustainable domestic financing mechanisms, including health insurance expansion and public–private partnerships, to reduce donor dependency and ensure long-term AMR response capacity.

## Conclusion

Effectively combating AMR in Africa requires a multidimensional approach that integrates technical, socioeconomic, and cultural strategies. A genuinely people-centred framework is essential to confront the underlying drivers of resistance, ranging from weak governance and inadequate education to gender inequities, poverty, and limited access to healthcare, water, and sanitation. Embedding AMR containment within universal health coverage frameworks and prioritising marginalised and vulnerable populations will ensure equitable access to essential medicines and reduce antimicrobial misuse. Community participation, inclusive partnerships, and the incorporation of local knowledge are critical for designing interventions that reflect lived realities and foster ownership. Evidence-based policies that draw on interdisciplinary research and explicitly integrate socioeconomic dimensions will enhance the sustainability and public legitimacy of AMR strategies. Addressing cross-cutting challenges, including climate change, agricultural antibiotic use, and human mobility, alongside AMR will further strengthen health security. Ultimately, a unified approach rooted in equity, collaboration, and contextual adaptability is vital for reducing AMR and building resilient, people-centred health systems across Africa.

## Data Availability

No data sets were generated or analyzed.
